# Predictors of cognitive impairment and its association with mortality in maintenance hemodialysis patients: A prospective follow-up study

**DOI:** 10.12669/pjms.40.5.7836

**Published:** 2024

**Authors:** Muhammad Anees, Muhammad Shahbaz Pervaiz, Samreen Aziz, Irfan Elahi

**Affiliations:** 1 Muhammad Anees, MBBS, FCPS, Head of Nephrology Department, King Edward Medical University, Mayo Hospital, Lahore, Pakistan; 2 Muhammad Shahbaz Pervaiz, MBBS, FCPS, Senior Registrar of Nephrology Department, King Edward Medical University, Mayo Hospital, Lahore, Pakistan; 3 Samreen Aziz, MBBS, Post Graduate Resident, Nephrology Department, King Edward Medical University, Mayo Hospital, Lahore, Pakistan; 4 Irfan Elahi, MBBS, FCPS, Assistant Professor of Nephrology Department, King Edward Medical University, Mayo Hospital, Lahore, Pakistan

**Keywords:** Cognitive impairment, Maintenance haemodialysis, Age, Gender, Diabetes mellitus

## Abstract

**Objective:**

To determine predictors of cognitive impairment (CI) and its association with mortality in maintenance haemodialysis (MHD) patients.

**Methods:**

This prospective follow up study was conducted at HD Department, Mayo Hospital, Lahore from September, 2021 to November, 2022. All patients undergoing MHD for more than three months and having age between 18-65 years were included while those with dialysis duration less than three months, history of neuropsychiatric illness was excluded. Cognitive function was assessed using British Columbia Cognitive Complaints Inventory (BC-CCI) questionnaire. CI was defined on Likert scale as mild, moderate, and severe. Patients were followed up to one year regarding outcome measures.

**Results:**

One hundred and four patients were included in the study. Mean age was 45.86±11.11 years and Diabetes Mellitus (DM) was the most common cause of End Stage Renal Disease (ESRD) in 39(37.5%) patients. CI was found in 86(82.7%) patients in following order of severity: mild 61(58.7%), moderate 19(18.3%) and severe 6(5.9%). Increasing age (≥50 years), gender (female), Diabetes Mellitus (DM), unemployment and education <10^th^ grade were found as significant predictors of CI (*p*<0.05). Significant positive correlations of CI score with age (r=0.338, *p*<0.001) and MHD duration (r=0.211, *p*=0.032) were found. However, the CI was not significantly associated with mortality (p=0.302).

**Conclusion:**

CI was common in MHD patients. Factors affecting CI were increasing age, female gender, DM, unemployment and low education level. CI was not associated with mortality in MHD patients.

## INTRODUCTION

Cognition is the mental process of acquiring knowledge and understanding through reasoning, experience and senses.^1^ The six well known cognitive domains are social, complex attention, language, memory-learning, perceptual motor and executive functions.^1^ The acquired deficit in one or more of these cognitive domains is termed as CI.^1^ CI is 2.5 times more prevalent in Chronic Kidney Disease (CKD) patients as compared to the general population (51% Vs. 19%).^2^,^3^ Moreover, the risk for CI increases by 11% for every 10ml/min/1.73sqm decline in Glomerular Filtration Rate (GFR).^4^ The prevalence of CI in patients undergoing MHD is markedly high (76%).^5^ CI in CKD is multifactorial with Cerebrovascular Disease (CVD) being its most predominant underlying cause.^6^,^7^ Other causative factors may include uremic toxins, anemia, depression, sleep disturbance, intradialytic hypotension, cerebral micro-embolisms or bleeds and electrolytes abnprmalities.^6^-^8^

The demographic and clinical factors associated with increased risk for CI in MHD patients are increasing age, female gender, low education level, Diabetes Mellitus(DM), Hypertension (HTN), vitamin D deficiency, secondary hyperparathyroidism, prolonged dialysis vintage and low single pool (sp) Kt/V.^6^-^9^ CI leads to reduced compliance with diet, medications and dialysis scheduling in these patients, worsening their quality of life (QOL), hospitalization and mortality.^7^ Local literature available^10^ on CI in MHD is scarce, so there is dire need to work on this important and neglected aspect of kidney disease patients. The objective of this study was to determine the predictors of CI and its association with mortality in MHD patients.

## METHODS

This prospective follow up study was conducted at the HD unit of Nephrology Department, Mayo Hospital, and Lahore from September, 2021 to November, 2022.

### Ethical Approval

It was obtained from the Institutional Review Board (IRB), King Edward Medical University, Lahore (No. 1081/RC/KEMU dated 27- 12-2022 in continuation of letter No.66/RC/KEMU dated 18-1-21).

All patients of CKD undergoing MHD for more than three months and having age between 18-65 years were included in the study while those with dialysis duration shorter than three months, pregnancy, severe anaemia (Haemoglobin(Hb) < 7.0 g/dl), Chronic Liver Disease (CLD), history of neuropsychiatric illness were excluded.CI was assessed during dialysis session applying Urdu version of BC-CCI after obtaining informed consent. BC-CCI is a rapid screening tool which consists of six items assessing perceived problems with concentration, memory, expressing thoughts, word finding, slow thinking, and difficulty solving problems in the past seven days.Patients were requested to respond according to their condition during the last one week. The questionnaire was administrated again to same patient after two weeks to get their response. The mean response values of two points were included in data analysis.

The cognitive status score was classified into normal (0-4), mild (5-9), moderate (10-14) and severe (15-18). Demographics and clinical data (cause of ESRD, MHD duration, average spKt/V, and HD frequency) of the patients were collected on a predesigned survey form. Blood samples were sent for haematological (Hb), biochemical parameters as serum urea, creatinine, sodium, potassium, calcium and phosphorus measurements. All patients were followed up to one year regarding their outcome measures. The data was entered and analysed using IBM-SPSS Ver-23 and Med Calc Ver -20.009. The continuous variables were expressed as Mean ± SD, whereas categorical variables in the form of frequencies and percentages. Relative Risk (RR) with 95% Confidence Interval was applied to find the significant predictors of CI. Pearson correlation coefficient was employed to investigate correlation between cognitive impairment score and other continuous variables. Kaplan Meier curve was used to display survival of the patients with CI. A *p* value < 0.05 was considered as statistically significant.

## RESULTS

**Fig.1:**
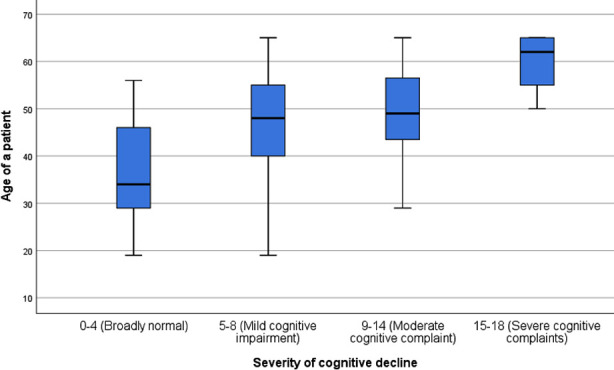
Box plot showing association between severity of CI and age of patients.

**Fig.2:**
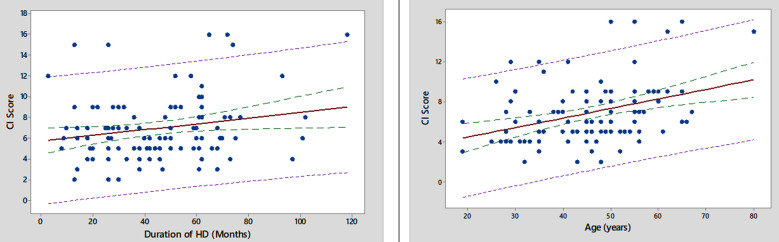
Scatter plot showing correlation between CI score MHD duration and Age.

**Fig.3:**
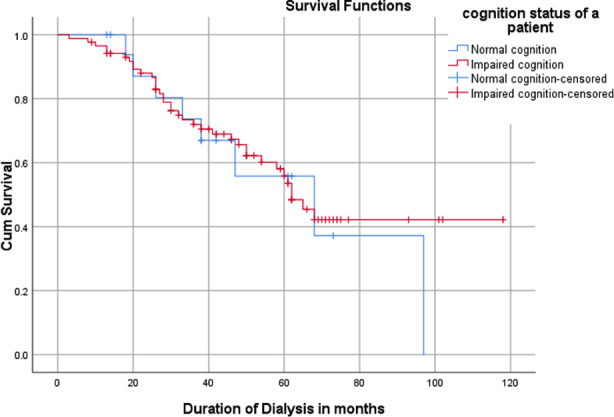
Kaplan Miere Survival curve of CI patients on MHD.

One hundred and four patients who fulfilled the inclusion criteria were enrolled. Mean age of patients was 45.86±11.11 years with majority of them 60(57.7%) were under 50 Years. Most of patients were male 59(56.7%), unemployed 81(77.9%) and with education of less than 10^th^ grade 57(54.8%). DM and HTN were the two most common causes of MHD in 39(37.5%) and 38(36.5%) patients respectively. Mean duration of MHD was 44.07±23.26 months and most of them 69(66.3%) had inadequate HD. On the average Hb level was 10.41±1.78 g/dl and hyperphosphatemia (PO_4_ > 4.5 mg/dl) were the most common electrolyte disorder observed in 81(77.9%) patients. CI was found in most 86(82.7%) of the patients with order of severity as mild 61(58.7%), moderate 19(18.3%) and severe 6(5.8%). Increasing age (*p*=0.001), female gender (*p*=0.008), DM (*p*=0.004), unemployment (*p*=0.012) and low education level (*p*=0.019) were found statistically significant predictors of CI (Table-I). Significant positive correlation of CI score was found with age of patient (r=0.338, *p*<0.001) and MHD duration (r=0.211, *p*=0.032) as displayed in Fig.1 and 2. At one year follow up, 85 patients remained alive and 19 patients were deceased. CI was not significantly associated with increased mortality (*p*=0.302). Kaplan-Meier survival curve did not display significant association between CI and mortality of patients as shown in Fig.3.

**Table-I:** RR of Demographics, Clinical and Laboratory parameters for CI (n=104).

Sr#	Parameters	Cognitive Status		RR	95% CI	p-value

		Impaired (n=86) %	Normal (n=18) %			
1	***Age (years)***					
	1. 18-49	44 (51.2)	16 (88.9)	1.30	1.10-1.53	0.001
	2. 50-65	42 (48.8)	2 (11.1)			
2	***Gender***					
	1. Male	44 (51.2)	15 (83.3)	1.25	1.05-1.48	0.008
	2. Female	42 (48.8)	3 (16.7)			
3	***Education (grades)***					
	1. < 10^th^	52 (60.4)	5 (27.8)	1.26	1.03-1.53	0.019
	2. ≥ 10^th^	34 (39.6)	13 (72.2)			
4	***Employment***					
	1. Yes	13 (15.1)	10 (55.5)	1.59	1.10-2.29	0.012
	2. No	73 (84.9)	8 (44.5)			
5	***Income (Rs.)***					
	1. ≤ 30,000	27 (31.4)	10 (55.5)	0.82	0.66-1.02	0.086
	2. > 30,000	59 (68.6)	8 (45.5)			
6	***Cause of ESRD***					
	1. Diabetic	37 (43)	2 (11.1)	1.25	1.07-1.47	0.004
	2. Non-Diabetic	49 (57)	16 (88.9)			
7	***Hb (g/dl)***					
	1. < 10	39 (45.3)	5 (27.8)	1.13	0.95-1.34	0.154
	2. ≥ 10	47 (54.7)	13 (72.2)			
8	***Serum Na (mmol/l)***					
	1. < 135	29 (33.7)	6 (33.3)	1.00	0.83-1.20	0.974
	2. ≥ 135	57 (66.3)	12 (66.7)			
9	***Ca×P (mg^2^/dl^2^)***					
	1. ≤ 55	61 (70.9)	10 (55.5)	0.88	0.71-1.09	0.250
	2. > 55	25 (29.1)	8 (45.5)			
10	***HD Duration (months)***					
	1. ≤ 60	60 (69.8)	13 (72.2)	1.02	0.84-1.23	0.779
	2. > 60	27 (30.2)	5 (27.8)			
11	***HD Frequency (/week)***					
	1. Twice	47 (54.7)	12 (66.7)	0.91	0.77-1.09	0.338
	2. Thrice	39 45.3)	6 (33.3)			
12	***Avg spKt/V***					
	1. < 1.2	41 (47.7)	5 (27.8)	1.14	0.96-1.36	0.112
	2. ≥ 1.2	45 (52.3)	13 (72.2)			
13	***Outcome Measures***					
	1.Alive	69(80.2)	16(88.9)	1.10	0.91-1.32	0.302
	2.Deceased	17 (19.8)	2 (11.1)			

## DISCUSSION

In this study, the incidence of CI in MHD patients was 82.7% which was comparable with previous study conducted in Pakistan.^10^ However this incidence was very high as compared with other Asian countries like Japan (18.8%)^11^, Iran (32%)^12^ and India (44%).^13^ Surprisingly, it was even higher than developing African countries like Ethopia where prevelance of CI in CKD patients was 49.1%.^14^ This difference may be due to demographic, social and assessment tool used for CI. Local dynamics of ESRD patients also play an exclusive role in CI. Nephrology services are in the early phase of development and there is limited number of nephrologist as compared to developed countries.^15^ Due to this, people and even medical professional are not well versed about the early referral of the CKD patients to nephrologist^15^ and present when they are just at the verge of dialysis. Late presentation causes longer exposure of the body to uremic toxins (phosphate, fibroblast growth factor 23(FGF 23), hyperhomocysteinemia, xanthine) which pass through the blood brain barrier and cause neurodegeneration.^16^ Late presentation not only causes high hospitalization rate but also increases mortality.^17^ Studies have shown if the load of uremic toxins is reduced by renal transplantation then CI is improved.^18^ Another factor affecting CI is the timing of taking the interview for assessment of CI.^19^ Recent medical literature shows that dialysis causes a sudden change in cerebral blood flow due to changes in ultrafiltration rate and volume causing CI.

In contrast to previous studies conducted locally^10^ and internationally^17^, demographic factors like younger age was amongst the main predictors of CI in MHD patients in our study. In this study, mean age of the patient was 45 years and CI was higher in patients with relatively younger age as compared to other studies where mean age is much higher.^9^ According to Karakizlis H et al,^17^ CI was 75% in patients with mean age of 71.6yeras. This situation is alarming for health authorities and nephrologists as well because CI is affecting younger population in Pakistan due to multiple reasons as already discussed. CI causes deficits in many brain functions like learning, memory and processing in patients with CKD with further progression of CKD and at the time of dialysis initiation, memory loss, difficulty in execution and language deficits become an important feature. All these aspects lead to poor QOL and unemployment in our patients as observed in this study. Unemployment is one of the important factors affecting CI in these patients and it may be vice versa. These patients are not only inactive member of society but they are also financial burden on their families. Due to poor status of health, patients are accompanied by one to two family members which puts the things further into disaster.

In present study, increasing age had positive correlation with cognitive impairment due to certain changes in brain. With advancing age, the brain undergoes several structural changes like cerebral atrophy, reduced hippocampal volume, thinning of cerebral cortex, with an effect on cognitive function.^20^ Cerebral atrophy is usually 0.5% per year with considerable variation which is accelerated in patients with hemodialysis. White matter hyperintensities represent small vessel diseases which are twice in dialysis population.^21^ CI among general population transpires predominantly in females.^22^ Women exhibits earlier and rapid cognitive decline with aging as compared to males from reduced synthesis of gonadal hormones.^19^

Majority of the female patients had CI in current study. In this male dominant society, health of the male is taken as priority because he is earning hand of the family and present early for medical management causing diminished CI than opposite gender. `While females are dependent on the family has lesser employment, lower income, poorer stress coping capabilities precipitating CI. It has been observed that poorly educated people commonly have CI because of limited cognitive reserves.^11^

Most of MHD patients in this study having CI had low education level which was not observed in local study^10^ but observed in previous international studies.^11^, ^13^ They don’t have an access to electronic and print media about the diseases with deprived lifestyle. As compared to educated people uneducated patients have limited knowledge about their disease, complications, preventions, its management and have poor compliance with diet, medications and dialysis schedule.

CI is five times more common in diabetics than general population. Diabetic patients having CKD are twice more susceptible to CI than CKD patients without diabetes^22^ as observed in this study. It has been observed locally that diabetic patients don’t take their disease very seriously and try to manage it with non-medical measures like herbal therapies. General population is not taking oral drugs regularly and they have fear about taking insulin causing uncontrolled diabetes (high HbA1C level). It has been observed that our diabetic patients have lot of complications like blindness, ischemic heart disease and stroke at a very younger age. This is due to neuronal malfunctioning from severe hypoglycemia, micro-angiopathy, oxidative stress and inflammation.^16^

Prolongation of dialysis therapy is associated with CI as observed in this and other studies.^9^,^10^ Contributing factors related to dialysis treatment including cerebral ischemia from intradialytic hypotension or micro-embolism, cerebral edema from electrolyte imbalance, cerebral micro-bleeds from improper anticoagulation technique and cerebrovascular disease. Moreover, advancing age, worsening depression, enhancing malnutrition, decreasing sleep, increasing drug side effects might have greater impact on cognition under prolonged dialysis setting.

Almost half of the patients were having low Hemoglobin (Hb) level in the current study. We did not find statistically significant correlation between Hb level and CI score but it was negatively correlated supporting that as Hb level falls CI score increases. Literature also supports that if anemia is improved with Erythropoietin (EPO), CI will be decreased.^23^

Our study was first study to determine association between CI and mortality in MHD patients in Pakistan. In this study, CI was not significantly associated with mortality as compared to previous studies.^24^,^25^ The possible explanation could be that patients with relatively younger age (mean age 45 ± 11 years) were included in our study as compared with other studies. Yidan Gau et al^24^ and Kunhao Bai et al.^25^ reported significant association between CI and mortality in older patients (mean age 63 ± 7 years^24^,80 years and above^25^) undergoing dialysis. The impact of CI on mortality in older individuals were due to increased risk of falls, multiple comorbidities, poor functional capacity, poor compliance to medical care due to their physical and financial dependence in these studies.

### Limitations

It includes small sample size and a single center study.

## CONCLUSION

CI was common in MHD patients. Factors affecting CI were increasing age, female gender, DM, unemployment and low education level. CI was not associated with mortality in MHD patients.

### Recommendations:

- Patients with CKD must be referred to Nephrologist at very early stage for creating awareness about future prognoses and making of AV Fistula.
- Early initiation of dialysis to avoid the exposure of brain to uremic toxins.
- Routine screening of the dialysis patients for CI.

### Authors’ Contributions:

**MA:** Conception and designing of the work, final approval of the version to be published and accountable for all aspects of the work.

**MSP:** Drafting the work and revising it for important intellectual content.

**SA:** Acquisition, analysis and interpretation of data.

**IE:** Literature review and contributions to designing of the work.
